# An Accelerating Reduction Approach for Incomplete Decision Table Using Positive Approximation Set

**DOI:** 10.3390/s22062211

**Published:** 2022-03-12

**Authors:** Tao Yan, Chongzhao Han, Kaitong Zhang, Chengnan Wang

**Affiliations:** 1 School of Electronic and Information Engineering, Xi’an Jiaotong University, Xi’an 710049, China; czhan@xjtu.edu.cn (C.H.); zkt0301@stu.xjtu.edu.cn (K.Z.); chengnanhz@stu.xjtu.edu.cn (C.W.); 2 MOE Key Lab for Intelligent Network and Network Security, Xi’an Jiaotong University, Xi’an 710049, China

**Keywords:** rough set, incomplete decision table, variable precision model, attribute reduction, positive approximation set

## Abstract

Due to the explosive growth of data collected by various sensors, it has become a difficult problem determining how to conduct feature selection more efficiently. To address this problem, we offer a fresh insight into rough set theory from the perspective of a positive approximation set. It is found that a granularity domain can be used to characterize the target knowledge, because of its form of a covering with respect to a tolerance relation. On the basis of this fact, a novel heuristic approach ARIPA is proposed to accelerate representative reduction algorithms for incomplete decision table. As a result, ARIPA in classical rough set model and ARIPA-IVPR in variable precision rough set model are realized respectively. Moreover, ARIPA is adopted to improve the computational efficiency of two existing state-of-the-art reduction algorithms. To demonstrate the effectiveness of the improved algorithms, a variety of experiments utilizing four UCI incomplete data sets are conducted. The performances of improved algorithms are compared with those of original ones as well. Numerical experiments justify that our accelerating approach enhances the existing algorithms to accomplish the reduction task more quickly. In some cases, they fulfill attribute reduction even more stably than the original algorithms do.

## 1. Introduction

With the wide usage of a diversity of advanced sensors, heterogeneous information acquisition in real-world applications has become much more simple. It also brings the challenge of dealing with a huge amount of data collected by these sensors and generating useful information. To address this challenge, multiple intelligent computing approaches were proposed, e.g., fuzzy set theory, Dempster–Shafer evidence theory, and rough set theory. Rough set theory (RST) is considered as a generalization of set theory for analyzing and processing a variety of data sets consisting of incomplete, imprecise, inconsistent, or uncertain data. It originated from Zdzislaw I. Pawlak [1] and has been identified as a creative and innovative mathematical tool in the last two decades. The rough-set-based data mining approaches have superiority in that they need no prior information, in contrast with other widely utilized strategies, such as SVM, PCA, and DNN [2,3,4,5,6]. Attribute reduction, or feature selection, has become one of the hot spots in the research area of big data. In recent years, the number of objects and dimensions of data sets has been increasing exponentially, as well as the quantity of large-scale data sets. For example, hundreds of thousands of attributes, which reflect various characteristics of the corresponding objects in practice, are stored in various data-sets [7]. However, a large portion of them give no benefit to the subsequent pattern recognition at all, but only take up precious storage space and consume computing time in vain. Hence, it has become a research focus to overcome this obstacle.

All conventional attribute reduction approaches can be classified into three main strategies—filtering, packing, and embedding [8]. The first strategy picks up attribute subsets on the basis of a specific type of measure, e.g., distance [9], information gain [10], dependence [11], and consistency [12]. There exist two types among these measures, one is based on distance and the other is based on consistency [13]. The second strategy adopts a particular learning algorithm to evaluate and choose attribute subsets. The third strategy is a combined strategy of the above two. Generally, the ultimate goal of rough-set-based attribute reduction is to make sure that the chosen attribute subset with lower dimension owns exactly the same discriminability as the universal set of original attributes, but does not maximize the discriminability of classes blindly [14].

The problem of attribute reduction has received increasing attention in recent decades and efforts have been made by different researchers to address various drawbacks. One of the representative methods is proposed by Skowron, who employed the discriminability matrix to retrieve all potential reducts from a given data set [15]. To fulfill the reduction task for an incomplete decision table (IDT), Skowron’s method was developed by Kryszkiewicz into a generalized approach utilizing discriminability matrix [16]. Shu et al. researched an incremental attribute selection approach for the data sets with dynamic incomplete data to improve the performances of other algorithms [17,18,19]. To evaluate candidate features in incomplete data, Qian and Shu studied a feature selection approach on the basis of mutual information criterion [20]. Jin and Li investigated in a reduction algorithm based on positive region, i.e., FPR algorithm, to reduce the computation load of attribute reduction [21]. Yan and Han presented an conditional entropy-based reduction algorithm for IDT to evaluate the uncertainty of condition attributes and eliminate redundant ones [22,23]. Xie and Qin investigated the inconsistency degree and demonstrated an incremental attribute reduction algorithm in dynamic data environments [24]. Ma et al. researched a general steg analysis attribute selection approach on the basis of α-positive region reduction [25]. Jing et al. introduced the incremental mechanisms of computing a reduct with a multi-granulation view and gave a method of updating reducts as the objects and attributes of DT change dynamically, or increasing simultaneously [26,27]. Sun et al. proposed an fuzzy neighborhood multi-granulation rough-set-based feature selection approach in neighborhood decision systems [28]. Unfortunately, some of the aforementioned methods and other reduction approaches can only deal with the issue of reduction for decision table, but not for IDT, because of the high complexity of the latter. Additionally, almost all of conventional reduction approaches for IDT would suffer from different degrees of long processing time due to large-scale computation when they process incomplete decision tables. To overcome this shortcoming, a variety of heuristic algorithms have been investigated, which can shorten computing time and reserve certain properties of corresponding IDT [29,30,31,32,33,34]. Nevertheless, their efficiencies for practical applications are still not satisfying. That is why we made our efforts to realize attribute reduction for IDT in a more intelligent and more efficient manner.

The aim of this paper is not to find a way of generating superior reducts, in contrast with most of existing attribute reduction approaches, but to study how to search for the same reduct in a more efficient way. Furthermore, the accelerating approaches for existing reduction algorithms in different rough set models, as well as their properties, are investigated in this paper. The major contributions of this research work are concluded as follows: (1) The concept of positive approximation set is constructed and one of its properties is investigated; (2) A novel heuristic accelerating approach of attribute reduction using positive approximation set for IDT is proposed; (3) The implementations of our accelerating approach are realized in different rough set models and tested by utilizing incomplete UCI data sets in the real world; (4) The performances of both computing time and stability of the proposed approach are exhibited and compared with some most recent reduction methods to verify its superiority. The simulations justify that our approach outputs precisely the same reducts as other reduction methods, while it consumes evidently less time and operates more stably in some cases. This paper is organized as follows. Some relevant preliminaries and background concepts are briefly reviewed in Section 2. The details of positive-approximation-set-based reduction approach are provided in Section 3. Section 4 conducts a series of simulations utilizing UCI data sets and gives some analysis. Section 5 draws some conclusions.

## 2. Preliminaries

For the purpose of presenting our accelerating approach, it is of significance to review some concepts of rough set concerning our main subject at the very beginning. The rough set theory was firstly proposed by Z. Pawlak to describe and tackle imprecise, uncertain, and vague concepts [1]. Both classical and generalized rough set model contain a variety of mathematical concepts and definitions. To keep our research understandable, some preliminaries are presented in this section at first. Additional mathematical foundations of this paper, described in more detail, with some examples, can be found in [22].

### 2.1. Classical Rough Set Model

RST-based attribute reduction begins with a given data table, i.e., an information system (IS). It consists of all objects we are interested in, as well as their features which can be described by a finite attribute set. An IS containing non-empty attribute values is considered as a complete IS, otherwise it implies as an incomplete information system (IIS). Generally, meeting empty attribute value in data mining and other data processing is almost inevitable. These empty values commonly stand for unavailable feature or inaccessible data, which may be caused by the error in measurement, the impreciseness in data acquisition, the low level of belief in the obtained data, and other potential factors. Therefore, an IIS means the existence of unavailable data or missing value in the system [35]. If an IIS contains a decision attribute which is different from other condition attributes and can indicate the category of the corresponding object, then it stands for an incomplete decision table (IDT).

An IS can be described by a pair $\left(U,A\right)$, where $U=\left\{x_{1},\ldots ,x_{n}\right\}$ indicates the universe of discourse which is actually a non-empty, finite set of objects, and $A=\left\{a_{1},\ldots ,a_{m}\right\}$ indicates a finite attribute set. There also exists a mapping $a:U\to V_{a}$ for any a∈A, where $V_{a}$ denotes the domain of the attribute *a*.

A decision table (DT) with the form of $\left(U,C\cup \left\{d\right\}\right)$ is actually a special information system, where *C* indicates the whole condition attribute set in DT which can reflect specific features of the target object, and d∉C indicates decision attribute which implies the object’s category. Let $V_{d}$ indicate the domain of decision attribute mapping $d\left(x\right)$. An attribute set is actually a feature set for pattern classification, and a training pattern set or its sign set can be represented by the universe of discourse.

Let ${\left[x\right]}_{R}$ denote an equivalence relation on *U*, and *∅* denote an empty set. It implies that relation *R* is reflexive, symmetric, and transitive. Hence it can generate a partition $U/R=IND\left(R\right)=\left\{{\left[x\right]}_{R}|x\in U\right\}$ on *U*, where $IND\left(R\right)$ indicates a equivalence class (i.e., an indiscernible class) which is generated by the relation *R*. In RST, it can also be considered as an elementary set of *R*. As for any target set X⊆U, the following two elementary sets of *R* can be used to approximate *X*.

(1) $$R_{-}\left(X\right)=\left\{{\left[x\right]}_{R}\left|{\left[x\right]}_{R}\subseteq X\right.\right\}$$

(2) $$R^{-}\left(X\right)=\left\{{\left[x\right]}_{R}\left|{\left[x\right]}_{R}\cap X\neq \emptyset \right.\right\}$$

They are defined as the lower and upper approximation sets of *X*, respectively. Furthermore, the equations of positive region, negative region, boundary region, and approximation measure are, respectively, presented as

(3) $$\mathrm{POS}\left(X\right)=R_{-}\left(X\right)$$

(4) $$\mathrm{NEG}\left(X\right)=U-R^{-}\left(X\right)$$

(5) $$\mathrm{BND}\left(X\right)=R^{-}\left(X\right)-R_{-}\left(X\right)$$

(6) $$\alpha_{R}\left(X\right)=\frac{\left|R_{-}\left(X\right)\right|}{\left|R^{-}\left(X\right)\right|}$$

where X≠∅. The lower approximation is equivalent with the positive region of *X*, which denotes a subset consisting of the objects that can be undoubtedly classified as members of *X*. In contrast, the upper approximation consists of the objects that are possibly members of *X*. Moreover, the negative region consists of the objects that can be definitely ruled out as the members of *X*. Finally, the approximation measure $\alpha_{R}\left(X\right)$ is utilized to evaluate the completeness degree of our knowledge on X.

We use ∗ to denote empty attribute value, which means that the value of the corresponding condition attribute of the object is missing or unavailable. The IS and DT containing ∗ attribute value are, respectively, defined as incomplete information system (IIS) and incomplete decision table (IDT). Commonly, the process of attribute reduction for incomplete data set is starts with an IDT.

### 2.2. Incomplete Variable Precision Rough Set Model

In the latest decade, a variety of generalized rough-set-model-based reduction approaches have been proposed and developed. This subsection is dedicated to introducing some notations concerning incomplete variable precision model for use.

Let $\left(U,A\right)$ be an IS which owns attribute subset P⊆A. The definition of a binary similarity relation on *U* can be expressed as

(7) $$\mathrm{SIM}\left(P\right)=\left\{\left(x,y\right)\in U\times U\left|\forall a\in P,\left[a\left(x\right)=a\left(y\right)\right]\cup \left[a\left(x\right)=*\right]\cup \left[a\left(y\right)=*\right]\right.\right\}$$

As a matter of fact, $\mathrm{SIM}\left(P\right)$ is essentially a tolerance relation on *P*. It can be simply obtained that $\mathrm{SIM}\left(P\right)=\cap_{a\in P}\mathrm{SIM}\left(\left\{a\right\}\right)$.

Let $\mathrm{SIM}\left(P\right)=\cap_{a\in P}\mathrm{SIM}\left(\left\{a\right\}\right)$ be an IIS, P⊆A be a subset of condition attributes *A*, and *X* be a subset of the universe of discourse *U*. The target set *X* can be approximated by $\bar{\mathrm{SIM}\left(P\right)}X$ and $\underline{\mathrm{SIM}\left(P\right)}X$, i.e.,

(8) SIMP_X=∪Y∈UUSIMPSIMPY⊆XSIMP¯X=∪Y∈UUSIMPSIMPY∩X≠∅

where UUSIMPSIMP denotes a partition of the universe of discourse *U* with respect to $\mathrm{SIM}\left(P\right)$.

A classification task for DT can be characterized by $\mathrm{DT}=\left(U,C\cup D\right)$, where *C* indicates the universe of condition attributes, *D* indicates the decision attribute set, and there exists C∩D=∅. All objects are assumed to be partitioned by *D* into *r* disjoint sets, i.e., $\left\{X_{1},X_{2},\ldots ,X_{r}\right\}$. Given a tolerance relation, $\mathrm{SIM}\left(P\right)$, generated from *P*, where *P* indicates a condition attribute subset P⊆C, then the lower and upper approximation set with respect to *D* can be defined, respectively, as

(9) $$\left\{\begin{array}{c} \underline{\mathrm{SIM}\left(P\right)}D\;=\;\left\{\underline{\mathrm{SIM}\left(P\right)}X_{1},\underline{\mathrm{SIM}\left(P\right)}X_{2},\ldots ,\underline{\mathrm{SIM}\left(P\right)}X_{r}\right\}\\ \bar{\mathrm{SIM}\left(P\right)}D\;=\;\left\{\bar{\mathrm{SIM}\left(P\right)}X_{1},\bar{\mathrm{SIM}\left(P\right)}X_{2},\ldots ,\bar{\mathrm{SIM}\left(P\right)}X_{r}\right\} \end{array}\right.$$

Given $POS_{P}\left(D\right)\;=\;{⋃}_{i=1}^{r}\underline{\mathrm{SIM}\left(P\right)}X_{i}$, i.e., the positive region of *D* with respect to *P*. The misclassification function *c* and the granularity-based approximation set have been proposed to construct variable precision rough set models [36]. This model can be further generalized for acquiring a more flexible algorithm for IDT attribute reduction.

Let the pair $\left(U,A\right)$ be an IIS, P⊆A be a subset of condition attributes, and *X* be a target subset of the universe of discourse *U*. The threshold β is given as $\beta \in \left[0,0.5\right]$, then *X* can be approximated by ${\underline{\mathrm{SIM}\left(P\right)}}^{\beta }X$ and ${\bar{\mathrm{SIM}\left(P\right)}}^{\beta }X$, i.e.,

(10) $$\left\{\begin{array}{c} {\underline{\mathrm{SIM}\left(P\right)}}^{\beta }X=\left\{x\left|D\left(S_{P}\left(x\right),S_{P}\left(x\right)\cap X\right)\leq \beta ,x\in X\right.\right\}\\ {\bar{\mathrm{SIM}\left(P\right)}}^{\beta }X=\left\{x\left|D\left(S_{P}\left(x\right),S_{P}\left(x\right)\cap X\right)\leq 1-\beta ,x\in U\right.\right\} \end{array}\right.$$

where they satisfy ${\underline{\mathrm{SIM}\left(P\right)}}^{\beta }X\subseteq X\subseteq {\bar{\mathrm{SIM}\left(P\right)}}^{\beta }X$.

Let the pair $\left(U,C\cup D\right)$ be a DT. All objects are assumed to be partitioned by *D* into *r* disjoint sets, i.e., $\left\{X_{1},X_{2},\ldots ,X_{r}\right\}$. Given a tolerance relation $\mathrm{SIM}\left(P\right)$ generated from *P*, where *P* indicates a condition attribute subset P⊆C, then the lower and upper approximation set with respect to *D* in variable precision model can be defined, respectively, as

(11) $$\left\{\;\;\;\;\begin{array}{c} \;\;{\underline{\mathrm{SIM}\left(P\right)}}^{\beta }D=\left\{{\underline{\mathrm{SIM}\left(P\right)}}^{\beta }X_{1},{\underline{\mathrm{SIM}\left(P\right)}}^{\beta }X_{2},\ldots ,{\underline{\mathrm{SIM}\left(P\right)}}^{\beta }X_{r}\right\}\\ \;\;{\bar{\mathrm{SIM}\left(P\right)}}^{\beta }D=\left\{{\bar{\mathrm{SIM}\left(P\right)}}^{\beta }X_{1},{\bar{\mathrm{SIM}\left(P\right)}}^{\beta }X_{2},\ldots ,{\bar{\mathrm{SIM}\left(P\right)}}^{\beta }X_{r}\right\} \end{array}\right.$$

The positive region of rough set in variable precision model can be obtained as $POS_{P}^{\beta }\left(D\right)={⋃}_{i=1}^{r}\underline{\mathrm{SIM}{\left(P\right)}^{\beta }}X_{i}$, i.e., β-positive region of *D* with respect to *P*. According to the above framework, a novel algorithm can be demonstrated for attribute reduction in an incomplete variable precision model.

Let the pair $\left(U,A\right)$ be an IIS. Given a partial order relation $\underline{≺}$ on $2^{A}$ (power set of *A*) [36], if set *P* is crisper than set *Q*, in other words *Q* is rougher than *P*, then it is definite that $P\underline{≺}Q$ satisfies (if $S_{P}\left(x_{i}\right)\subseteq S_{Q}\left(x_{i}\right)$ holds for any $i\in \left\{1,2,\ldots ,\left|U\right|\right\}$). If P≠Q and $P\underline{≺}Q$ satisfy simultaneously, then we use the notation P≺Q.

### 2.3. The Positive Approximation Set of IIS and IDT

An introduction of positive approximation set is demonstrated in this subsection as a preparation for proposing our algorithm. With regard to an incomplete data set, a granularity domain, which can be employed to describe target knowledge, is provided by a covering generated from a tolerance relation. Furthermore, a sequence of granularity domains ranging from rough to crisp is determined by a corresponding sequence of condition attribute subsets with granularity (same ranging as the domains) in the power set of condition attributes.

Let the pair $\left(U,A\right)$ be an IIS, X⊆U be a target subset, and $P=\left\{P_{1},P_{2},\ldots ,P_{n}\right\}$ be a subset family satisfying $P_{1}\underline{≻}P_{2}\underline{≻}\ldots \underline{≻}P_{n}$, where $P_{i}\in 2^{A}$, i=1,2,…,n. Given $P_{i}=\left\{P_{1},P_{2},\ldots ,P_{i}\right\}$, $P_{i}$-lower and $P_{i}$-upper approximation sets of *X* for IIS can be defined as

(12) $$\left\{\begin{array}{c} \underline{P_{i}}\left(X\right)=\overset{i}{\underset{k=1}{⋃}}\underline{\mathrm{SIM}\left(P_{k}\right)}X_{k}\\ \bar{P_{i}}\left(X\right)=\bar{\mathrm{SIM}\left(P_{i}\right)}X \end{array}\right.$$

where $X_{1}=X$. It can be obtained that $X_{k}=X-{⋃}_{j=1}^{k-1}\underline{P_{j}}\left(X_{j}\right)$ for k=2,3,…,i, where i=1,2,…,n. This definition demonstrates the fact that *X* can be approximated by the corresponding approximation sets, i.e., $\underline{P_{i}}\left(X\right)$ and $\bar{P_{i}}\left(X\right)$. The $P_{i}$-lower and $P_{i}$-upper approximation sets of *X* for IIS in variable precision model can be defined, respectively, as

(13) $$\left\{\begin{array}{c} {\underline{P_{i}}}^{\beta }\left(X\right)=\overset{i}{\underset{k=1}{⋃}}{\underline{\mathrm{SIM}\left(P_{k}\right)}}^{\beta }X_{k}\\ {\bar{P_{i}}}^{\beta }\left(X\right)={\bar{\mathrm{SIM}\left(P_{i}\right)}}^{\beta }X \end{array}\right.$$

Let the pair $\left(U,A\right)$ be an IIS, X⊆U be a target subset, and $P=\left\{P_{1},P_{2},\ldots ,P_{n}\right\}$ be a subset family satisfying $P_{1}\underline{≻}P_{2}\underline{≻}\ldots \underline{≻}P_{n}$, where $P_{i}\in 2^{A}$, i=1,2,…,n. Given $P_{i}=\left\{P_{1},P_{2},\ldots ,P_{i}\right\}$, where i=1,2,…,n, it can be obtained that

(14) $${\mathrm{POS}}_{P_{i+1}}^{U}\left(D\right)={\mathrm{POS}}_{P_{i}}^{U}\left(D\right)\cup {\mathrm{POS}}_{P_{i+1}}^{U_{i+1}}\left(D\right)$$

where $U_{1}=U$, $U_{i+1}=U-{\mathrm{POS}}_{P_{i}}^{U}\left(D\right)$. Since the positive approximation set of IIS is related to the structure of target concept *X* (i.e. it is related to the tolerance class in the lower approximation set of *X* with respect to P), the tolerance class on *U* can be employed to redefine the P-positive approximation set of *X*.

Let the pair $\left(U,C\cup D\right)$ be an IDT, X⊆U be a target subset, $P=\left\{P_{1},P_{2},\ldots ,P_{n}\right\}$ be a subset family satisfying $P_{1}\underline{≻}P_{2}\underline{≻}\ldots \underline{≻}P_{n}$, and UUDD=X1,X2,…,Xr be a partition of the universe, *U*, with respect to *D*. The P-lower and P-upper approximation sets of *D* for IDT can be defined, respectively, as

(15) $$\left\{\begin{array}{c} \underline{P}D=\left\{\underline{P}\left(X_{1}\right),\underline{P}\left(X_{2}\right),\ldots ,\underline{P}\left(X_{r}\right)\right\}\\ \bar{P}D=\left\{\bar{P}\left(X_{1}\right),\bar{P}\left(X_{2}\right),\ldots ,\bar{P}\left(X_{r}\right)\right\} \end{array}\right.$$

The P-lower and P-upper approximation sets of *D* for IDT in a variable precision model can be defined, respectively, as

(16) $$\left\{\begin{array}{c} {\underline{P}}^{\beta }D=\left\{{\underline{P}}^{\beta }\left(X_{1}\right),{\underline{P}}^{\beta }\left(X_{2}\right),\ldots ,{\underline{P}}^{\beta }\left(X_{r}\right)\right\}\\ {\bar{P}}^{\beta }D=\left\{{\bar{P}}^{\beta }\left(X_{1}\right),{\bar{P}}^{\beta }\left(X_{2}\right),\ldots ,{\bar{P}}^{\beta }\left(X_{r}\right)\right\} \end{array}\right.$$

There exists a similar conclusion for IDT, which is ${\mathrm{POS}}_{P_{i+1}}^{\beta U}\left(D\right)={\mathrm{POS}}_{P_{i}}^{\beta U}\left(D\right)\cup {\mathrm{POS}}_{P_{i+1}}^{\beta U_{i+1}}\left(D\right)$, where $U_{1}=U$, $U_{i+1}=U-{\mathrm{POS}}_{P_{i}}^{\beta U}\left(D\right)$. This implies that the granularity sequence can be used to approximate the target knowledge *D* from positive direction. Our accelerating reduction algorithm for IDT was mainly inspired by this conclusion.

## 3. Accelerating Reduction Approach for IDT Using Positive Approximation Set

To achieve the ultimate goal of attribute reduction, it is necessary to obtain the specific attribute subset that contains least condition attributes and reserve the same discriminability as *C*. Three procedures should be taken into consideration for realizing a heuristic reduction algorithm—searching strategy, significance evaluation, and termination condition.

Most of conventional heuristic algorithms for attribute reduction have been suffering from huge amounts of computation in different degree. For addressing this disadvantage, our research does not intend to design a brand new reduction algorithm directly, but to utilize the aforementioned positive approximation set to optimize the existing heuristic strategies for reduction and improve their performances.

### 3.1. Definitions of Condition Attribute Significance

One of modern reduction approaches proposed by Xie et al. (abbreviation as IPR) [24] is adopted in the following section. It is essentially developed from Shu’s algorithm [18,19]. To realize our accelerating reduction algorithm, several definitions of condition attribute significance should be presented at first. Each of the these definitions can be utilized for the subsequent reduction process.

**Definition** **1.** *Let the pair $\left(U,C\cup D\right)$ be an IDT, and B⊆C be a subset of condition attributes. As for ∀a∈B, the definition of the condition attribute significance of a inside B can be expressed as*

(17) $${\mathrm{SIG}}_{1}^{\mathrm{inner}}\left(a,B,D\right)=\gamma_{B}\left(D\right)-\gamma_{B-\left\{a\right\}}\left(D\right)$$

*where γBD=POSBDPOSBDUU.*

**Definition** **2.** *Let the pair $\left(U,C\cup D\right)$ be an IDT, and B⊆C be a subset of condition attributes. As for ∀a∈C−B, the definition of the condition attribute significance of a outside B can be expressed as*

(18) $${\mathrm{SIG}}_{1}^{\mathrm{outer}}\left(a,B,D\right)=\gamma_{B\cup \left\{a\right\}}\left(D\right)-\gamma_{B}\left(D\right)$$

The above two definitions are provided by Qian and Liang et al. [37], and the following two come from Liang and Shi et al. [35].

**Definition** **3.** *Let the pair $\left(U,C\cup D\right)$ be an IDT, and B⊆C be a subset of condition attributes. As for ∀a∈B, the definition of the condition attribute significance of a inside B can be expressed as*

(19) $${\mathrm{SIG}}_{2}^{\mathrm{inner}}\left(a,B,D\right)=E\left(D\left|B-\left\{a\right\}\right.\right)-E\left(D\left|B\right.\right)$$

**Definition** **4.** *Let the pair $\left(U,C\cup D\right)$ be an IDT, and B⊆C be a subset of condition attributes. As for ∀a∈C−B, the definition of the condition attribute significance of a outside B can be expressed as*

(20) $${\mathrm{SIG}}_{2}^{\mathrm{outer}}\left(a,B,D\right)=E\left(D\left|B\right.\right)-E\left(D\left|B\cup \left\{a\right\}\right.\right)$$

On the basis of Definitions 1 and 2, the corresponding measures of significance can be utilized to construct a new algorithm in incomplete variable precision model, which is capable of reserving β-positive region with respect to the target knowledge *D*.

**Definition** **5.** *Let the pair $\left(U,C\cup D\right)$ be an IDT, and B⊆C be a subset of condition attributes. As for ∀a∈B, the definition of the condition attribute significance of a inside B can be expressed as*

(21) $${\mathrm{SIG}}_{3}^{\mathrm{inner}}\left(a,B,D\right)=\gamma_{B}^{\beta }\left(D\right)-\gamma_{B-\left\{a\right\}}^{\beta }\left(D\right)$$

*where γBβD=POSBβDPOSBβDUU.*

**Definition** **6.** *Let the pair $\left(U,C\cup D\right)$ be an IDT, and B⊆C be a subset of condition attributes. As for ∀a∈C−B, the definition of the condition attribute significance of a outside B can be expressed as*

(22) $${\mathrm{SIG}}_{3}^{\mathrm{outer}}\left(a,B,D\right)=\gamma_{B\cup \left\{a\right\}}^{\beta }\left(D\right)-\gamma_{B}^{\beta }\left(D\right)$$

### 3.2. Rank Reservation Property of Attribute Significance

This subsection plans to give a discussion on rank reservation property of the condition attribute significance to provide a theory fundamental for proposing our accelerating reduction algorithm. For simplicity and clarity of the content, the notation ${\mathrm{SIG}}_{\lambda }^{\mathrm{outer}}\left(a,U,B,D\right)$ is adopted to indicate the condition attribute significance in previous subsection, where $\lambda \in \left(1,2,3\right)$. Additionally, $S_{B}^{U}\left(x\right)$ denotes a tolerance class generated from the object *x*, with respect to the attribute subset *B*, on the universe of discourse *U*. The detailed proofs of all lemmas and theorems appearing in this subsection are demonstrated in Appendix A and Appendix B , respectively.

Firstly, two Lemmas are presented and proved aiming at investigating in the rank reservation property of the dependence based condition attribute significance for IDT.

**Lemma** **1.** *Let $A,B,C,A^{\prime },B^{\prime },C^{\prime }$ be six finite set such that we have $A^{\prime }=A\cup C$ and $B^{\prime }=B\cup C^{\prime }$ satisfied. If $A^{\prime }\subseteq B^{\prime }$ and $C^{\prime }\cap \left(A\cup B\right)=\emptyset$ satisfy, then we have A⊆B.*

**Lemma** **2.** *Let the pair $\left(U,C\cup D\right)$ be an IDT, such that we have B⊆C and $U^{\prime }=U-{\mathrm{POS}}_{B}^{U}\left(D\right)$ satisfied. If $S_{B\cup \left\{a\right\}}^{U}\left(x^{\prime }\right)\subseteq S_{D}^{U}\left(x^{\prime }\right)$ and $x^{\prime }\in U^{\prime }$ satisfy, then we have $S_{B\cup \left\{a\right\}}^{U^{\prime }}\left(x^{\prime }\right)\subseteq S_{D}^{U^{\prime }}\left(x^{\prime }\right)$.*

Secondly, the theorem of rank reservation property can be proved as follows according to Lemmas 1 and 2.

**Theorem** **1.** *Let the pair $\left(U,C\cup D\right)$ be an IDT, such that we have B⊆C and $U^{\prime }=U-{\mathrm{POS}}_{B}^{U}\left(D\right)$ satisfied. As for ∀a,b∈C−B, if ${\mathrm{SIG}}_{1}^{\mathrm{outer}}\left(a,U,B,D\right)\geq {\mathrm{SIG}}_{1}^{\mathrm{outer}}\left(b,U,B,D\right)$ is satisfied, then we have*

(23) $${\mathrm{SIG}}_{1}^{\mathrm{outer}}\left(a,U^{\prime },B,D\right)\geq {\mathrm{SIG}}_{1}^{\mathrm{outer}}\left(b,U^{\prime },B,D\right)$$

Finally, to investigate in the rank reservation property of condition attribute significance in Yan’s conditional entropy reduction approach for IDT [23], the following Lemma 3 is indispensable. Additionally, this property can be described by Theorems 2 and 3 in incomplete rough set model and incomplete variable precision model, respectively.

**Lemma** **3.** *Let the pair $\left(U,C\cup D\right)$ be an IDT, such that we have B⊆C and $U^{\prime }=U-{\mathrm{POS}}_{B}^{U}\left(D\right)$ satisfied. Then, we have $\left|S_{B}^{U}\left(x^{\prime }\right)\right|-\left|S_{B}^{U}\left(x^{\prime }\right)\cap S_{D}^{U}\left(x^{\prime }\right)\right|=\left|S_{B}^{U^{\prime }}\left(x^{\prime }\right)\right|-\left|S_{B}^{U^{\prime }}\left(x^{\prime }\right)\cap S_{D}^{U^{\prime }}\left(x^{\prime }\right)\right|$, where $x^{\prime }\in U^{\prime }$.*

**Theorem** **2.** *Let the pair $\left(U,C\cup D\right)$ be an IDT, such that we have B⊆C and $U^{\prime }=U-{\mathrm{POS}}_{B}^{U}\left(D\right)$ satisfied. As for ∀a,b∈C−B, if ${\mathrm{SIG}}_{2}^{\mathrm{outer}}\left(a,U,B,D\right)\geq {\mathrm{SIG}}_{2}^{\mathrm{outer}}\left(b,U,B,D\right)$, then there exists ${\mathrm{SIG}}_{2}^{\mathrm{outer}}\left(a,U^{\prime },B,D\right)\geq {\mathrm{SIG}}_{2}^{\mathrm{outer}}\left(b,U^{\prime },B,D\right)$.*

**Theorem** **3.** *Let the pair $\left(U,C\cup D\right)$ be an IDT, such that we have B⊆C, β=0 and $U^{\prime }=U-{\mathrm{POS}}_{B}^{U}\left(D\right)$ satisfied. As for ∀a,b∈C−B, if ${\mathrm{SIG}}_{3}^{\mathrm{outer}}\left(a,U,B,D\right)\geq {\mathrm{SIG}}_{3}^{\mathrm{outer}}\left(b,U,B,D\right)$ satisfies, then there exists ${\mathrm{SIG}}_{3}^{\mathrm{outer}}\left(a,U^{\prime },B,D\right)\geq {\mathrm{SIG}}_{3}^{\mathrm{outer}}\left(b,U^{\prime },B,D\right)$.*

It can be concluded from the above theorems that the result of reduction would be unchanged as the object number of lower approximation set of positive approximation set for IDT is reduced. In other words, the significance rank of the selected reducts can be reserved when the positive region of positive approximation set for IDT narrows.

### 3.3. Accelerating Attribute Reduction Algorithms

Generally, all reduction approaches based on RST are designed to find a minimal subset consisting of no redundant attribute and reserving specific property, like the whole universe of condition attributes *C*. It is essentially NP-hard to seek out all potential reducts of an IDT, hence it is only necessary to search for any of them.

It is indispensable to achieve the tolerance class generated from the concerning attributes. Therefore, an accelerating algorithm of tolerance class acquisition for IDT reduction is proposed. The inspiration of this implementation partially comes from the method of radix sorting, and the computation complexity of the algorithm equates as follows:

(24) $$O\;\;\left(\;\;\left|A\right|\;\left|U\right|\;\;+\;\;\sum_{j=1}^{\left|A\right|}\;\;\sum_{k=1}^{j-1}\;\;\left|{*}_{a_{k}}\right|\;\left|V_{a_{k}}\right|\;\;\right)\;\;\approx \;\;O\;\;\left(\left|A\right|\;\left|U\right|\;\;+\;\;{\left|A\right|}^{2}\;\left|U\right|\right)\;\;=\;\;O\;\;\left({\left|A\right|}^{2}\;\left|U\right|\right)$$

where $\left|{*}_{a_{k}}\right|$ indicates the number of objects that own empty value in condition attribute $a_{k}$, and $\left|V_{a_{k}}\right|$ indicates the number of objects that own no empty value in $a_{k}$. A derived result of reduced computation complexity equates $O\;\;\left({\left|A\right|}^{2}\;\left|U\right|\right)$.

The analysis of computation complexity reveals that the dimension of condition attributes has greater influence in the length of computing time, compared with the amount of target objects. Based on the above discussion, an accelerating reduction approach for IDT using positive approximation set (ARIPA) is proposed. In the framework of ARIPA, the evaluation function (or termination condition) can be expressed as ${\mathrm{EF}}^{U}\left(B,D\right)={\mathrm{EF}}^{U}\left(C,D\right)$, which implies that the discernibility of condition attribute subset *B* is exactly the same as that of the universe of condition attributes *C*. The evaluation function can be chosen according to the original reduction algorithm we plan to accelerate. For an instance, if the original algorithm adopted is Yan’s rough conditional entropy-based reduction algorithm in [23], then the corresponding evaluation function should be ${\mathrm{EN}}^{U}\left(B,D\right)={\mathrm{EN}}^{U}\left(C,D\right)$, where EN denotes the rough conditional entropy. In other words, if ${\mathrm{EF}}^{U}\left(B,D\right)={\mathrm{EF}}^{U}\left(C,D\right)$ satisfies, then *B* should be one of the reducts we search for. The detailed steps of ARIPA are exhibited as follows. The outer significance ${\mathrm{SIG}}^{\mathrm{outer}}\left(a_{k},red,D,U_{i}\right)$ and inner significance ${\mathrm{SIG}}^{\mathrm{inner}}\left(a_{k},C,D,U\right)$ in Algorithm 1 can be either the pair of ${\mathrm{SIG}}_{1}^{\mathrm{outer}}$, ${\mathrm{SIG}}_{1}^{\mathrm{inner}}$ or the pair of ${\mathrm{SIG}}_{2}^{\mathrm{outer}}$, ${\mathrm{SIG}}_{2}^{\mathrm{inner}}$.    
**Algorithm 1: **ARIPA.**Input: **$\mathrm{IDT}=\left(U,C\cup D\right)$**Output:** Attribute reduct red1: Initialize red as *∅*, i.e., red←∅, where red indicates   condition attribute subset which has been selected.2: Evaluate ${\mathrm{SIG}}^{\mathrm{inner}}\left(a_{k},C,D,U\right)$, where $k\leq \left|C\right|$.3: **If** ${\mathrm{SIG}}^{\mathrm{inner}}\left(a_{k},C,D,U\right)>0$, **then** add $a_{k}$ into red.   IDT’s kernel partly consists of condition attributes   in red at this step.4: i←1, $U_{1}\leftarrow U$, $R_{1}=red$, $P_{1}=\left\{R_{1}\right\}$.5: **While** $U_{i}\neq \emptyset$ and ${\mathrm{EF}}^{U_{i}}\left(red,D\right)\neq {\mathrm{EF}}^{U_{i}}\left(C,D\right)$, **do**6:       **{**Evaluate the positive region of the positive         approximation set ${\mathrm{POS}}_{P_{i}}^{U}\left(D\right)$,7:       $U_{i}=U-{\mathrm{POS}}_{P_{i}}^{U}\left(D\right)$,8:       i←i+1,9:       $red\leftarrow red\cup \left\{a_{0}\right\}$, where         ${\mathrm{SIG}}^{\mathrm{outer}}\left(a_{0},red,D,U_{i}\right)=\max\left\{{\mathrm{SIG}}^{\mathrm{outer}}\left(a_{k},red,D,U_{i}\right)\right\}$         $a_{k}\in C-red$**}**, **End**.10: $R_{i}\leftarrow R_{i}\cup \left\{a_{0}\right\},P_{i}\leftarrow \left\{R_{1},R_{2},\ldots ,R_{i}\right\}$.11: Return red.

    To accelerate the reduction algorithm in the incomplete variable precision rough set (IVPR) model by ARIPA, it is remodeled on the basis of the β-positive approximation set. The IVPR-version of accelerating reduction, Algorithm 2 (ARIPA-IVPR), is illustrated as follows.   
**Algorithm 2: **ARIPA-IVPR.**Input: **$\mathrm{IDT}=\left(U,C\cup D\right)$, threshold β≤0.5**Output:** Attribute reduct red1: Initialize red as *∅*, i.e., red←∅, where red indicates   condition attribute subset which has been selected.2: Evaluate ${\mathrm{SIG}}_{3}^{\mathrm{inner}}\left(a_{k},C,D,U\right)$, where $k\leq \left|C\right|$.3: **If** ${\mathrm{SIG}}_{3}^{\mathrm{inner}}\left(a_{k},C,D,U\right)>0$, **then** add $a_{k}$ into red.   IDT’s kernel partly consists of condition attributes   in red at this step.4: i←1, $U_{1}\leftarrow U$, $R_{1}=red$, $P_{1}=\left\{R_{1}\right\}$.5: **While** $U_{i}\neq \emptyset$ and $\gamma_{red}^{\beta U_{i}}\left(D\right)\neq \gamma_{C}^{\beta U_{i}}\left(D\right)$, **do**6:       **{**Evaluate the positive region of the positive         approximation set ${\mathrm{POS}}_{P_{i}}^{\beta U}\left(D\right)$,7:       $U_{i}=U-{\mathrm{POS}}_{P_{i}}^{\beta U}\left(D\right)$,8:       i←i+1,9:       $red\leftarrow red\cup \left\{a_{0}\right\}$, where         ${\mathrm{SIG}}_{3}^{\mathrm{outer}}\left(a_{0},red,D,U_{i}\right)=\max\left\{{\mathrm{SIG}}_{3}^{\mathrm{outer}}\left(a_{k},red,D,U_{i}\right)\right\}$         $a_{k}\in C-red$**}**, **End**.10: $R_{i}\leftarrow R_{i}\cup \left\{a_{0}\right\},P_{i}\leftarrow \left\{R_{1},R_{2},\ldots ,R_{i}\right\}$.11: Return red.

## 4. Experiments

To investigate the efficiency and effectiveness of the proposed ARIPA and ARIPA-IVPR, four incomplete data sets are picked up from the UCI Machine Learning Database at University of California for experimental purposes. The performances of the proposed algorithms were analyzed and compared with those of other state-of-the-art algorithms to prove their superiority.

### 4.1. Experiments on ARIPA and ARIPA-IVPR

Due to the existence of continuous attribute values contained in the chosen incomplete data sets, Tsai’s CACC discretization algorithm [38] is adopted as a preprocess before reduction to discretize continuous values into discrete ones. Another aim of this step is to reduce the computation load of subsequent steps and compress the data scale. The average CPU time of ARIPA, ARIPA-IVPR, and their competitors is counted in seconds as their running time. All simulation work is conducted on the PC with the configurations of 8GB RAM, Intel i5-8400 2.8GHz CPU, Matlab R2019a, Win10 (64 bit). The statistical results of the four incomplete data sets for simulations are summarized and analyzed, respectively, in Table 1.

**Figure 1:**
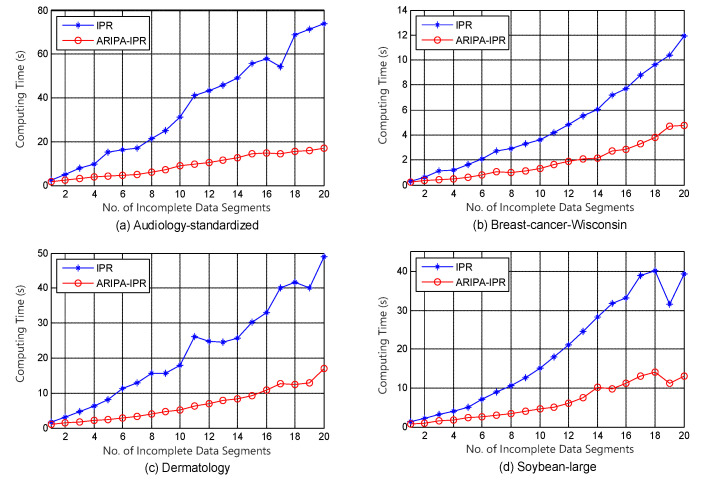
Computing time of IPR and ARIPA-IPR for (**a**) Audiology standardized, (**b**) Breast cancer Wisconsin, (**c**) Dermatology, and (**d**) Soybean large.

**Figure 2:**
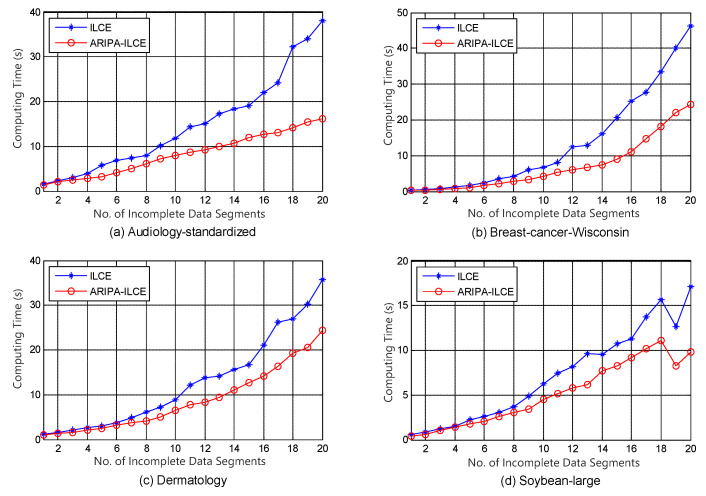
Computing time of ILCE and ARIPA-ILCE for (**a**) Audiology standardized, (**b**) Breast cancer Wisconsin, (**c**) Dermatology, and (**d**) Soybean large.

To compare our improved reduction algorithms with other competitors (Xie’s IPR [24] and Yan’s ILCE [23]), a modern approach is carried out for evaluating their computation complexities [39]. The same reduct would be obtained by each pair of the improved and original algorithm, thus we just have to make an comparison between their running times. The graphical illustrations of their performances are shown in Figure 1 and Figure 2. In these figures, the *x*-axis indicates the number of data segments which increases from 1 to 20 (all objects of each incomplete data set are equally divided into 20 segments), and the *y*-axis indicates the corresponding running time. The experiments using incomplete data segments in different scales would make us aware of the trend of the computing time as the scale grows. Furthermore, the simulations indirectly prove that our accelerating algorithm would exhibit more outstanding performance when the incomplete data set contains tens of thousands of objects.

**Figure 3:**
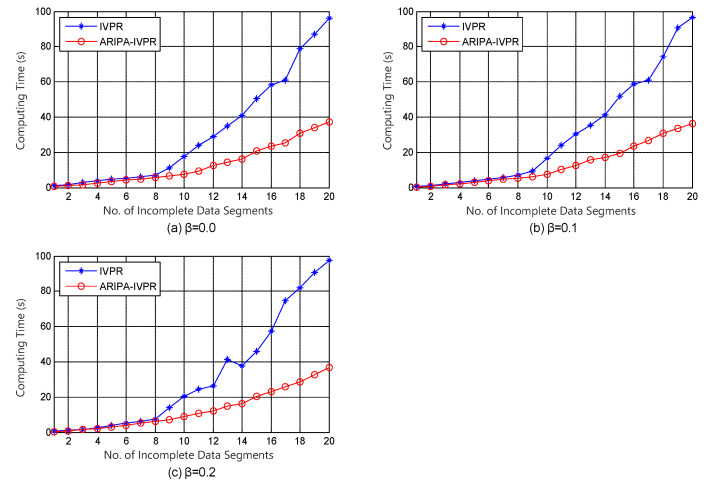
Computing time of IVPR and ARIPA-IVPR for Audiology standardized data set in (**a**) β=0.0, (**b**) β=0.1, and (**c**) β=0.2.

**Figure 4:**
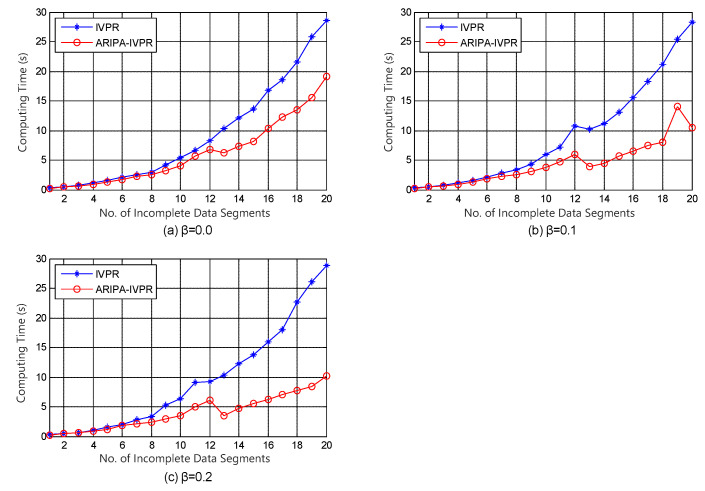
Computing time of IVPR and ARIPA-IVPR for Breast cancer Wisconsin data set in (**a**) β=0.0, (**b**) β=0.1, and (**c**) β=0.2.

**Figure 5:**
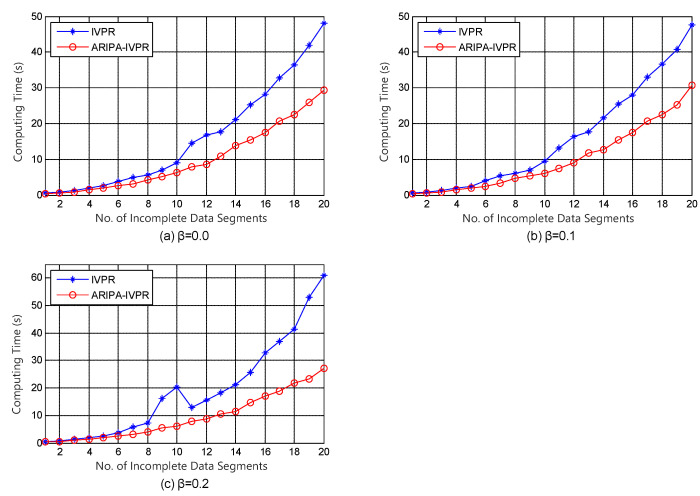
Computing time of IVPR and ARIPA-IVPR for Dermatology data set in (**a**) β=0.0, (**b**) β=0.1, and (**c**) β=0.2.

**Figure 6:**
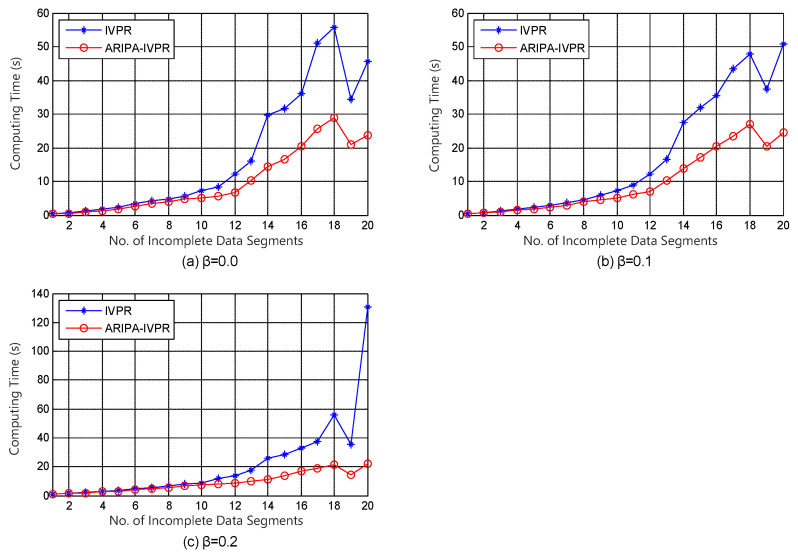
Computing time of IVPR and ARIPA-IVPR for Soybean large data set in (**a**) β=0.0, (**b**) β=0.1, and (**c**) β=0.2.

With regard to the framework of incomplete variable precision model, Kang’s IVPR algorithm [36] is conducted as a competitor for our improved ARIPA-IVPR. The experiment results are illustrated in Figure 3, Figure 4, Figure 5 and Figure 6.

### 4.2. Results and Discussions

It can be noticed from Figure 1, Figure 2, Figure 3, Figure 4, Figure 5 and Figure 6 that the computing time of the improved algorithm increases more smoothly than that of the original algorithm as the number of data segments grow. Essentially, this consequence can be the result for the following three reasons. (1) The accelerated algorithm consumes much less computing time when the universe of discourse shrinks dramatically. (2) As for the same incomplete data segments, the original algorithms have to consume more time to evaluate the condition attribute significance of the potential reducts. (3) Our accelerating algorithm would encapsulate all concerning objects into the lower approximation set with respect to the decision attribute set during the reduction, hence it ensures that the improved reduction algorithm would consume less time to finish the reduction. These results are caused by the rank reservation property of the condition attribute significance, as discussed in Section 3.2. It provides a solution to the inefficiency of the existing heuristic algorithms for IDT reduction. Since the reducts from different algorithms are identical, the same classification accuracy can be ensured in subsequent process, no matter what type of classifier is chosen, e.g., SVM, decision tree, etc. It is possible that the accelerating reduction algorithm we propose leads in the problem of over-fitting, in the perspective of classifier. However, discussion on this issue is not included in this paper.

It also can be observed that the computing time rises up for most of time when the number of data segments increases in each experiment, no matter which incomplete data set, competitor algorithm, style of rough set model, or value of β we choose. However, not all the curves show a strictly monotone increasing function, and the opposite may take place in a few cases (e.g., in Figure 4). This phenomenon a result of the possibility that the new added data segment, in contrast to the existing ones, may contain specific knowledge that is more useful for attribute reduction as well as compressing the computation load.

**Figure 7:**
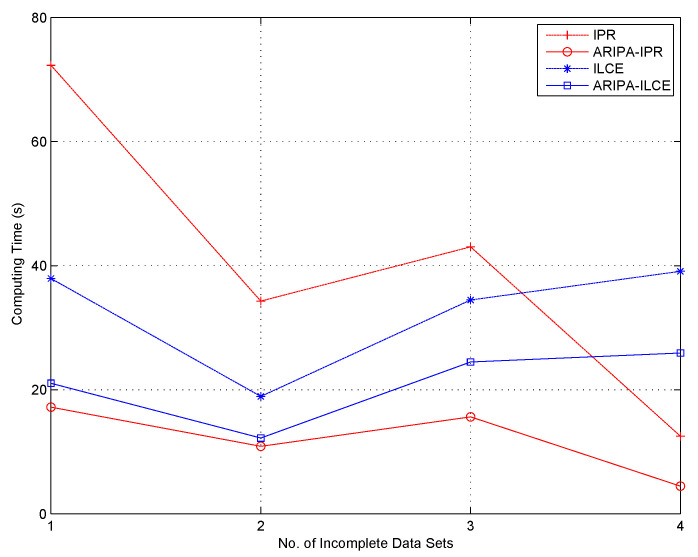
Computing time of IPR, ARIPA-IPR, ILCE, and ARIPA-ILCE for four incomplete data sets. (1—Audiology standardized; 2—Breast cancer Wisconsin; 3—Dermatology; 4—Soybean large.)

**Figure 8:**
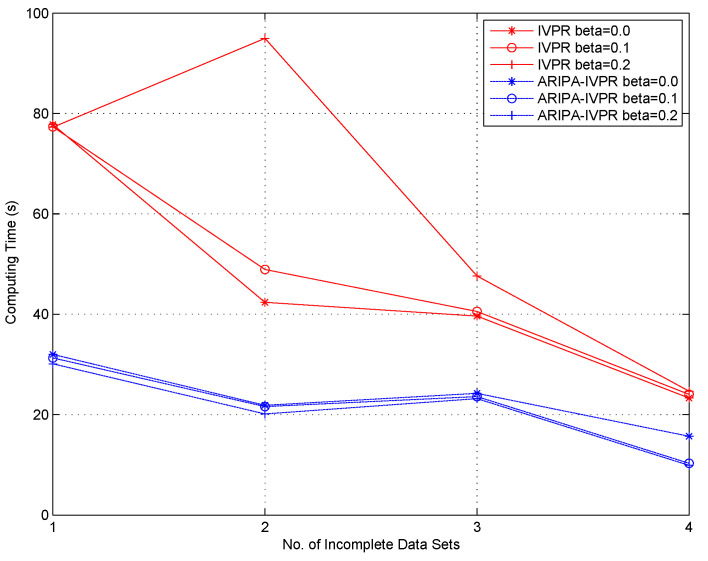
Computing time of IVPR and ARIPA-IVPR for four incomplete data sets (β=0.0,0.1,0.2). (1—Audiology standardized; 2—Breast cancer Wisconsin; 3—Dermatology; 4—Soybean large.)

The computation complexities of state-of-the-art [23] and improved algorithms are analyzed step by step in Table 2. It can be observed that the major difference in computation aspect is brought by step 2 and steps 5–9 of the algorithms. Among these steps, step 2 corresponds to the evaluation of the attribute significance of potential reducts, and steps 5–9 correspond to the loop which includes the evaluation of the positive region of positive approximation set and the heuristic search for real reducts. Moreover, Figure 7 and Figure 8 indicate that our improved algorithms run more efficiently than the original algorithms, both in rough set model and variable precision model (β = 0.0, 0.1, 0.2). Hence, the experiment results justify the conclusion that the accelerated algorithms are more efficient for reduction in practical applications.

### 4.3. Algorithm Stability Analysis

To evaluate the stability of both original and improved algorithms, ten-fold cross-validation was applied. In this validation, a given data set is randomly parted into ten nearly equally sized subsets. Nine of them are treated as training sets, and one last subset is reserved as a testing set to evaluate the classification accuracy. The distance between two different reducts $C_{i}$ and $C_{j}$ is evaluated in Equation (25), where $C_{0}$ and $C_{i}$ indicate the reducts generated from *U* and the *i*th segment of *U*, respectively.

(25) $$\mathrm{Distance}\left(C_{i},C_{j}\right)=1-\frac{\left|C_{i}⋂C_{j}\right|}{\left|C_{i}⋃C_{j}\right|}$$

Furthermore, by using the statistical method, mean (i.e., μ in Equation (26)) and standard deviation (i.e., σ in Equation (27)) of the above ten distances of the segments can be determined as well.

(26) $$\mu =\frac{1}{10}\sum_{i=1}^{10}\left(1-\frac{\left|C_{i}⋂C_{0}\right|}{\left|C_{i}⋃C_{0}\right|}\right)$$

(27) $$\sigma =\sqrt{\frac{1}{10}\sum_{i=1}^{10}{\left(\mathrm{Distance}\left(C_{i},C_{0}\right)-\mu \right)}^{2}}$$

The stability of the reduct outputted from the heuristic reduction algorithm is characterized by standard deviation of those distances. More specifically, lower the standard deviation gets, more stably the corresponding reduction algorithm would run. The stability analysis of each pair of algorithms is carried out in Table 3, Table 4 and Table 5.

In Table 3, it can be found that ARIPA-IPR consumes less computing time, and its lower standard deviation of computing time (in ten-fold cross-validation) implies better robustness than that of the original IPR algorithm. On the other hand, they both own exactly the same stability, as well as the same standard deviation of stability. By borrowing the positive approximation set approach, ARIPA-IPR not only reduces the computation of IPR evidently and enhances its robustness simultaneously, but also holds the same stability as IPR by generating the identical reduct. Similarly, same conclusions can be drawn from Table 4 for the pair of ARIPA-ILCE and ILCE. With regard to the pair of ARIPA-IVPR and IVPR in Table 5, the former half of the above conclusion still holds, and the stability of them are identical if β = 0.0. This result can be explained reasonably by Theorem 3. While in case of β = 0.1 or 0.2, ARIPA-IVPR runs more stably than IVPR does. This is because in incomplete variable precision rough set model, the selected reduct (which is with respect to a nonzero β), would become closer to the reduct generated from the universe of condition attributes, when the norm of the lower approximation set of the positive approximation set decreases.

When β varies between 0.0 and 0.5, it can be noticed that the reducts output from our reduction algorithm may be diverse in different cases. This result can be explained through the definition of incomplete variable precision model, i.e., the concerning inclusion degree function is non-monotonic. Although this does not meet our expectation, it is still meaningful because of the following reasons. (1) When the improved reduction algorithm meets its termination condition, the output reduct would definitely contain all the condition attributes that are included in the reduct output from the original reduction algorithm, on the condition that the compressed subset of universe $U_{i}$ is nonempty. Since the termination condition demands that $\gamma_{red}^{\beta U_{i}}\left(D\right)=\gamma_{C}^{\beta U_{i}}\left(D\right)$ and $\gamma_{red}^{\beta U}\left(D\right)=\gamma_{C}^{\beta U}\left(D\right)$ satisfy simultaneously, both of the reducts output from the original algorithm and the improved algorithm have the same approximation ability. (2) When the compressed subset of universe $U_{i}$ is empty, the dependence of the selected subset outputted from the improved algorithm would be γBβD=POSBβDPOSBβDUU=1. Since all of the objects in the universe of discourse *U* are encapsulated into the lower approximation set with respect to the decision attribute in this case, the improved reduction algorithm, which provides us with a more satisfying option, would have a better approximation capability than the original one.

## 5. Conclusions

To address the disadvantage of conventional methods of attribute reduction for incomplete decision table in the aspect of computational efficiency, the concept of a positive approximation set based on a tolerance relation is introduced. Additionally, the rank reservation property of the condition attribute significance is discussed, and it is employed to accelerate other existing reduction algorithms under various heuristic strategies. As a result, a novel accelerating reduction approach for IDT using positive approximation set (ARIPA) is proposed. Several state-of-the-art reduction algorithms in different rough set models are accelerated by ARIPA. To assess the performances of both improved and original reduction algorithms, a series of experiments utilizing four real-world incomplete data sets are conducted. The results show that the ARIPA-improved algorithm would ensure the output of the same reduct as that from the original reduction algorithm. While the former can finish attribute reduction in a more efficient and maybe a more stable manner, in contrast with the latter. Average computing time of ARIPA-IPR, ARIPA-ILCE, and ARIPA-IVPR is cut to 33.32%, 55.21%, and 43.62%, respectively. The proposed approach has been verified distinctly effective for dealing with incomplete data sets with large amounts of objects. However, the question of how to ensure its high efficiency for incomplete data sets with hundreds of thousands of dimensions (condition attributes) is still an unresolved issue left for the future.

## Figures and Tables

**Table 1:** Summary of the experimental incomplete data sets.

Incomplete Data Sets	Objects	Condition Attributes	Empty Values	Decision Classes	Incomplete Rate (%)
Audiology standardized	226	69	291	24	1.87
Breast cancer Wisconsin	699	10	16	2	0.23
Dermatology	366	34	8	6	0.06
Soybean large	307	35	712	19	6.63

**Table 2:** Analysis on the computation complexity of existing and accelerated attribute reduction algorithm.

Algorithms	Step 2	Step 3	Steps 5–9	Other Steps
Existing algorithm	$O\left({\left|C\right|}^{2}{\left|U\right|}^{2}\right)$	$O\left(\left|C\right|\right)$	$O\left(\sum_{i=1}^{\left|C\right|}{\left(\left|C\right|-i+1\right)}^{2}{\left|U\right|}^{2}\right)$	Constant
Accelerated algorithm	$\begin{array}{c} O\left({\left|C\right|}^{2}\left|U\right|+\left|C\right|\right.\\ \left.\sum_{j=1}^{\left|C\right|}\sum_{k=1}^{j-1}\left|{*}_{a_{k}}\right|\left|V_{a_{k}}\right|\right) \end{array}$	$O\left(\left|C\right|\right)$	$\begin{array}{c} O\left(\sum_{i=1}^{\left|C\right|}{\left(\left|C\right|-i+1\right)}^{2}\left|U_{i}\right|\right.+\\ \left.\left(\left|C\right|-i+1\right)\sum_{j=1}^{\left|C\right|-i+1}\sum_{k=1}^{j-1}\left|{*}_{a_{k}}^{U_{i}}\right|\left|V_{a_{k}}^{U_{i}}\right|\right) \end{array}$	Constant

**Table 3:** Computing time and stability of IPR and ARIPA-IPR for four incomplete data sets.

Incomplete Data Sets	IPR’s Computing Time (s)	ARIPA-IPR’s Computing Time (s)	IPR’s Stability	ARIPA-IPR’s Stability
Audiology standardized	71.5631±5.1558	17.0331±1.1149	0.2624±0.1380	0.2624±0.1380
Breast-cancer-WI	13.3757±1.6881	4.5208±0.8269	0.0792±0.1635	0.0792±0.1635
Dermatology	42.5979±1.9307	15.3719±0.5125	0.2893±0.2271	0.2893±0.2271
Soybean large	35.9553±3.9234	10.7832±1.4693	0.2289±0.2049	0.2289±0.2049

**Table 4:** Computing time and stability of ILCE and ARIPA-ILCE for four incomplete data sets.

Incomplete Data Sets	ILCE’s Computing Time (s)	ARIPA-ILCE’s Computing Time (s)	ILCE’s Stability	ARIPA-ILCE’s Stability
Audiology standardized	37.5503±2.7268	20.8380±1.1990	0.1868±0.1061	0.1868±0.1061
Breast cancer Wisconsin	38.6970±3.0960	25.6673±2.4608	0.0727±0.1160	0.0727±0.1160
Dermatology	34.1226±0.5987	24.2288±0.5979	0.2537±0.1784	0.2535±0.1784
Soybean large	18.7405±1.9096	12.1051±0.7328	0.1754±0.1349	0.1754±0.1349

**Table 5:** Computing time and stability of IVPR and ARIPA-IVPR for four incomplete data sets.

Incomplete Data Sets	β	IVPR’s Computing Time (s)	ARIPA-IVPR’s Computing Time (s)	IVPR’s Stability	ARIPA-IVPR’s Stability
Audiology standardized	0.0	76.4555±3.0168	31.0325±1.6524	0.2570±0.1351	0.2570±0.1351
	0.1	76.0280±3.5442	31.3581±1.5407	0.2356±0.1364	0.1782±0.0895
	0.2	75.9417±3.6175	29.2186±1.0746	0.2329±0.1705	0.1903±0.1479
Breast cancer Wisconsin	0.0	22.6102±3.1587	15.2200±3.1775	0.0678±0.1493	0.0678±0.1493
	0.1	23.3089±4.1228	9.9927±3.5050	0.1167±0.1779	0.0710±0.1136
	0.2	23.9333±3.6824	9.5895±3.6140	0.2272±0.2118	0.1389±0.2826
Dermatology	0.0	38.1813±0.3769	23.5210±0.4671	0.2209±0.1930	0.2209±0.1930
	0.1	39.3397±0.9589	22.8845±0.4153	0.3329±0.1241	0.2451±0.1771
	0.2	46.1933±5.9338	22.4904±0.3651	0.4899±0.2778	0.3640±0.1962
Soybean large	0.0	41.1054±5.1171	21.1961±2.9962	0.3520±0.1881	0.3520±0.1881
	0.1	47.4468±18.8684	20.9551±2.4125	0.3752±0.2183	0.3580±0.2016
	0.2	92.1092±23.7040	19.5275±2.0451	0.4695±0.1330	0.2348±0.1209

## Data Availability

Simulation data sets supporting reported results can be found at the link to publicly archived UCI Machine Learning Repository http://archive.ics.uci.edu/ml/index.php (accessed on 6 January 2022).

## Abbreviations

The following abbreviations are used in this manuscript:

RST	rough set theory
IS	information system
IIS	incomplete information system
DT	decision table
IDT	incomplete decision table
POS	positive region
NEG	negative region
BND	boundary region
SIM	similarity relation
SIG	condition attribute significance

## Appendix A

**Lemma** **A1.**
*Let $A,B,C,A^{\prime },B^{\prime },C^{\prime }$ be six finite set, such that we have $A^{\prime }=A\cup C$ and $B^{\prime }=B\cup C^{\prime }$ satisfied. If $A^{\prime }\subseteq B^{\prime }$ and $\left(A\cup B\right)\cap C^{\prime }=\emptyset$ satisfy, then we have A⊆B.*

**Proof.** Let a∈A satisfy, since $A^{\prime }=A\cup C$ and $A\subseteq A^{\prime }$, we have $a\in A^{\prime }$. Since $A^{\prime }\subseteq B^{\prime }$, we can derive $a\in B^{\prime }$. $\left(A\cup B\right)\cap C^{\prime }=\emptyset$, thus we have $A\cap C^{\prime }=\emptyset$; furthermore, $a\notin C^{\prime }$. Since $B^{\prime }=B\cup C^{\prime }$, $a\in B^{\prime }$ and $a\notin C^{\prime }$, there exists a∈B. Finally, we can obtain A⊆B. QED. □

**Lemma** **A2.**
*Let the pair $\left(U,C\cup D\right)$ be an IDT, such that we have B⊆C and $U^{\prime }=U-{\mathrm{POS}}_{B}^{U}\left(D\right)$ satisfied. If $S_{B\cup \left\{a\right\}}^{U}\left(x^{\prime }\right)\subseteq S_{D}^{U}\left(x^{\prime }\right)$ and $x^{\prime }\in U^{\prime }$ satisfy, then we have $S_{B\cup \left\{a\right\}}^{U^{\prime }}\left(x^{\prime }\right)\subseteq S_{D}^{U^{\prime }}\left(x^{\prime }\right)$.*

**Proof.** Since $U^{\prime }=U-{\mathrm{POS}}_{B}^{U}\left(D\right)$ and $x^{\prime }\in U^{\prime }$ satisfy, two notations *X* and *Y* can be defined as follows.

$$\begin{array}{cc} X & =\left\{x\left|\left(x\in S_{B\cup \left\{a\right\}}^{U}\left(x^{\prime }\right)\right)\cap \left(x\in {\mathrm{POS}}_{B}^{U}\left(D\right)\right)\right.\right\}\\ Y & =\left\{y\left|\left(y\in S_{D}^{U}\left(x^{\prime }\right)\right)\cap \left(y\in {\mathrm{POS}}_{B}^{U}\left(D\right)\right)\right.\right\} \end{array}$$

Therefore, we can obtain $S_{B\cup \left\{a\right\}}^{U}\left(x^{\prime }\right)=S_{B\cup \left\{a\right\}}^{U^{\prime }}\left(x^{\prime }\right)\cup X$ and $S_{D}^{U}\left(x^{\prime }\right)=S_{D}^{U^{\prime }}\left(x^{\prime }\right)\cup Y$. According to *Y*’s formula, it can be derived that $Y\subseteq {\mathrm{POS}}_{B}^{U}\left(D\right)$. Thus, there exists $Y\cap S_{B\cup \left\{a\right\}}^{U^{\prime }}\left(x^{\prime }\right)=\emptyset$ and $Y\cap S_{D}^{U^{\prime }}\left(x^{\prime }\right)=\emptyset$, i.e., $Y\cap \left(S_{B\cup \left\{a\right\}}^{U^{\prime }}\left(x^{\prime }\right)\cup S_{D}^{U^{\prime }}\left(x^{\prime }\right)\right)=\emptyset$. Then, according to $S_{B\cup \left\{a\right\}}^{U}\left(x^{\prime }\right)\subseteq S_{D}^{U}\left(x^{\prime }\right)$ and Lemma 1, it can be derived that $S_{B\cup \left\{a\right\}}^{U^{\prime }}\left(x^{\prime }\right)\subseteq S_{D}^{U^{\prime }}\left(x^{\prime }\right)$. □

**Lemma** **A3.**
*Let the pair $\left(U,C\cup D\right)$ be an IDT, such that we have B⊆C and $U^{\prime }=U-{\mathrm{POS}}_{B}^{U}\left(D\right)$ satisfied. Then, we have $\left|S_{B}^{U}\left(x^{\prime }\right)\right|-\left|S_{B}^{U}\left(x^{\prime }\right)\cap S_{D}^{U}\left(x^{\prime }\right)\right|=\left|S_{B}^{U^{\prime }}\left(x^{\prime }\right)\right|-\left|S_{B}^{U^{\prime }}\left(x^{\prime }\right)\cap S_{D}^{U^{\prime }}\left(x^{\prime }\right)\right|$, where $x^{\prime }\in U^{\prime }$.*

**Proof.** Since $U^{\prime }=U-{\mathrm{POS}}_{B}^{U}\left(D\right)$ and $x^{\prime }\in U^{\prime }$, two notations *X* and *Y* can be defined as follows.

$$\begin{array}{cc} X & =\left\{x\left|\left(x\in S_{B}^{U}\left(x^{\prime }\right)\right)\cap \left(x\in {\mathrm{POS}}_{B}^{U}\left(D\right)\right)\right.\right\}\\ Y & =\left\{y\left|\left(y\in S_{D}^{U}\left(x^{\prime }\right)\right)\cap \left(y\in {\mathrm{POS}}_{B}^{U}\left(D\right)\right)\right.\right\} \end{array}$$

Then it can be obtained that $S_{B}^{U}\left(x^{\prime }\right)=S_{B}^{U^{\prime }}\left(x^{\prime }\right)\cup X$ and $S_{D}^{U}\left(x^{\prime }\right)=S_{D}^{U^{\prime }}\left(x^{\prime }\right)\cup Y$. According to the definitions of *X* and *Y*, we can derive that $X\subseteq {\mathrm{POS}}_{B}^{U}\left(D\right)$ and $Y\subseteq {\mathrm{POS}}_{B}^{U}\left(D\right)$. Thus, there exists $Y\cap S_{B}^{U^{\prime }}\left(x^{\prime }\right)=\emptyset$ and $X\cap S_{D}^{U^{\prime }}\left(x^{\prime }\right)=\emptyset$. As for ∀x∈X, it can be obtained that $x\in S_{B}^{U}\left(x^{\prime }\right)$. It can be derived that $x^{\prime }\in S_{B}^{U}\left(x\right)$ on the basis of the symmetry of tolerance relation. Furthermore, it can be derived that $S_{B}^{U}\left(x\right)\subseteq S_{D}^{U}\left(x\right)$ on the basis of the definition of positive region, hence $x^{\prime }\in S_{D}^{U}\left(x\right)$. Similarly, it can be obtained that $x\in S_{D}^{U}\left(x^{\prime }\right)$. Since for ∀x∈X and $\forall x\in {\mathrm{POS}}_{B}^{U}\left(D\right)$, there exists x∈Y, i.e., X⊆Y. Therefore, the following formula can be derived.

$$\begin{array}{cc} S_{B}^{U}\left(x^{\prime }\right)\cap S_{D}^{U}\left(x^{\prime }\right) & =\left(S_{B}^{U^{\prime }}\left(x^{\prime }\right)\cup X\right)\cap \left(S_{D}^{U^{\prime }}\left(x^{\prime }\right)\cup Y\right)\\ \; & =\left(S_{B}^{U^{\prime }}\left(x^{\prime }\right)\cap S_{D}^{U^{\prime }}\left(x^{\prime }\right)\right)\cup \left(S_{B}^{U^{\prime }}\left(x^{\prime }\right)\cap Y\right)\cup \left(X\cap S_{D}^{U^{\prime }}\left(x^{\prime }\right)\right)\cup \left(X\cap Y\right)\\ \; & =\left(S_{B}^{U^{\prime }}\left(x^{\prime }\right)\cap S_{D}^{U^{\prime }}\left(x^{\prime }\right)\right)\cup X \end{array}$$

And since $X\subseteq {\mathrm{POS}}_{B}^{U}\left(D\right)$, we can obtain $\left(S_{B}^{U^{\prime }}\left(x^{\prime }\right)\cap S_{D}^{U^{\prime }}\left(x^{\prime }\right)\right)\cap X=\emptyset$ and $\left|S_{B}^{U}\left(x^{\prime }\right)\cap S_{D}^{U}\left(x^{\prime }\right)\right|=\left|S_{B}^{U^{\prime }}\left(x^{\prime }\right)\cap S_{D}^{U^{\prime }}\left(x^{\prime }\right)\right|+\left|X\right|$. Therefore, the following formula can be derived.

$$\begin{array}{cc} \left|S_{B}^{U^{\prime }}\left(x^{\prime }\right)\right|-\left|S_{B}^{U^{\prime }}\left(x^{\prime }\right)\cap S_{D}^{U^{\prime }}\left(x^{\prime }\right)\right| & =\left(\left|S_{B}^{U}\left(x^{\prime }\right)\right|-\left|X\right|\right)-\left(\left|S_{B}^{U}\left(x^{\prime }\right)\cap S_{D}^{U}\left(x^{\prime }\right)\right|-\left|X\right|\right)\\ \; & =\left|S_{B}^{U}\left(x^{\prime }\right)\right|-\left|X\right|-\left|S_{B}^{U}\left(x^{\prime }\right)\cap S_{D}^{U}\left(x^{\prime }\right)\right|+\left|X\right|\\ \; & =\left|S_{B}^{U}\left(x^{\prime }\right)\right|-\left|S_{B}^{U}\left(x^{\prime }\right)\cap S_{D}^{U}\left(x^{\prime }\right)\right| \end{array}$$

i.e., $\left|S_{B}^{U}\left(x^{\prime }\right)\right|-\left|S_{B}^{U}\left(x^{\prime }\right)\cap S_{D}^{U}\left(x^{\prime }\right)\right|=\left|S_{B}^{U^{\prime }}\left(x^{\prime }\right)\right|-\left|S_{B}^{U^{\prime }}\left(x^{\prime }\right)\cap S_{D}^{U^{\prime }}\left(x^{\prime }\right)\right|$, where $x^{\prime }\in U^{\prime }$. □

## Appendix B

**Theorem** **A1.**
*Let the pair $\left(U,C\cup D\right)$ be an IDT, such that we have B⊆C and $U^{\prime }=U-{\mathrm{POS}}_{B}^{U}\left(D\right)$ satisfied. As for ∀a,b∈C−B, if ${\mathrm{SIG}}_{1}^{\mathrm{outer}}\left(a,U,B,D\right)\geq {\mathrm{SIG}}_{1}^{\mathrm{outer}}\left(b,U,B,D\right)$ satisfies, then we have*

$${\mathrm{SIG}}_{1}^{\mathrm{outer}}\left(a,U^{\prime },B,D\right)\geq {\mathrm{SIG}}_{1}^{\mathrm{outer}}\left(b,U^{\prime },B,D\right).$$

**Proof.** According to the definition ${\mathrm{SIG}}_{1}^{\mathrm{outer}}\left(a,B,D\right)=\gamma_{B\cup \left\{a\right\}}\left(D\right)-\gamma_{B}\left(D\right)$, it is definite that the value of ${\mathrm{SIG}}_{1}^{\mathrm{outer}}\left(a,B,D\right)$ relies on the dependence function γBD=POSBDPOSBDUU. $U^{\prime }=U-{\mathrm{POS}}_{B}^{U}\left(D\right)$, hence, we can obtain ${\mathrm{POS}}_{B}^{U^{\prime }}\left(D\right)=\emptyset$. According to Lemma 2, we can obtain that if $S_{B\cup \left\{a\right\}}^{U}\left(x^{\prime }\right)\subseteq S_{D}^{U}\left(x^{\prime }\right)$, $x^{\prime }\in U^{\prime }$ satisfies, then there exists $S_{B\cup \left\{a\right\}}^{U^{\prime }}\left(x^{\prime }\right)\subseteq S_{D}^{U^{\prime }}\left(x^{\prime }\right)$. Therefore, it can be derived that ${\mathrm{POS}}_{B\cup \left\{a\right\}}^{U^{\prime }}\left(D\right)={\mathrm{POS}}_{B\cup \left\{a\right\}}^{U}\left(D\right)-{\mathrm{POS}}_{B}^{U}\left(D\right)$. Furthermore, we can the following:

$$\begin{array}{cc} \frac{{\mathrm{SIG}}_{1}^{\mathrm{outer}}\left(a,U,B,D\right)}{{\mathrm{SIG}}_{1}^{\mathrm{outer}}\left(a,U^{\prime },B,D\right)} & =\frac{\gamma_{B\cup \left\{a\right\}}^{U}\left(D\right)-\gamma_{B}^{U}\left(D\right)}{\gamma_{B\cup \left\{a\right\}}^{U^{\prime }}\left(D\right)-\gamma_{B}^{U^{\prime }}\left(D\right)}\\ \; & =\frac{\left|U^{\prime }\right|}{\left|U\right|}\frac{\left|{\mathrm{POS}}_{B\cup \left\{a\right\}}^{U}\left(D\right)\right|-\left|{\mathrm{POS}}_{B}^{U}\left(D\right)\right|}{\left|{\mathrm{POS}}_{B\cup \left\{a\right\}}^{U^{\prime }}\left(D\right)\right|-\left|{\mathrm{POS}}_{B}^{U^{\prime }}\left(D\right)\right|}\\ \; & =\frac{\left|U^{\prime }\right|}{\left|U\right|}\frac{\left|{\mathrm{POS}}_{B\cup \left\{a\right\}}^{U}\left(D\right)\right|-\left|{\mathrm{POS}}_{B}^{U}\left(D\right)\right|}{\left|{\mathrm{POS}}_{B\cup \left\{a\right\}}^{U}\left(D\right)\right|-\left|{\mathrm{POS}}_{B}^{U}\left(D\right)\right|}\\ \; & =\frac{\left|U^{\prime }\right|}{\left|U\right|}. \end{array}$$

Since 1≥U′U′UU≥0, then, if ${\mathrm{SIG}}_{1}^{\mathrm{outer}}\left(a,U,B,D\right)>{\mathrm{SIG}}_{1}^{\mathrm{outer}}\left(b,U,B,D\right)$ satisfies, then there exists ${\mathrm{SIG}}_{1}^{\mathrm{outer}}\left(a,U^{\prime },B,D\right)>{\mathrm{SIG}}_{1}^{\mathrm{outer}}\left(b,U^{\prime },B,D\right)$. □

**Theorem** **A2.**
*Let the pair $\left(U,C\cup D\right)$ be an IDT, such that we have B⊆C and $U^{\prime }=U-{\mathrm{POS}}_{B}^{U}\left(D\right)$ satisfied. As for ∀a,b∈C−B, if ${\mathrm{SIG}}_{2}^{\mathrm{outer}}\left(a,U,B,D\right)\geq {\mathrm{SIG}}_{2}^{\mathrm{outer}}\left(b,U,B,D\right)$, then there exists*

$${\mathrm{SIG}}_{2}^{\mathrm{outer}}\left(a,U^{\prime },B,D\right)\geq {\mathrm{SIG}}_{2}^{\mathrm{outer}}\left(b,U^{\prime },B,D\right).$$

**Proof.** Let UUSIMBSIMB=SBUx1,SBUx2,…,SBUxq,SBUxq+1,…,SBUxU and UUSIMDSIMD=SDUx1,SDUx2,…,SDUxq,SDUxq+1,…,SDUxU be true, where $x_{i}\in {\mathrm{POS}}_{B}^{U}\left(D\right)$, i=1,2,…,q. The notation ${\mathrm{EN}}^{U}\left(D\left|B\right.\right)$ is used to indicate the rough conditional entropy on the universe of discourse *U* in Yan’s approach [23].

$$\begin{array}{cc} {\mathrm{EN}}^{U}\left(D\left|B\right.\right) & =\frac{1}{{\left|U\right|}^{2}}\sum_{i=1}^{\left|U\right|}\left(\left|S_{B}^{U}\left(x_{i}\right)\right|-\left|S_{B}^{U}\left(x_{i}\right)\cap S_{D}^{U}\left(x_{i}\right)\right|\right)\\ \; & =\frac{1}{{\left|U\right|}^{2}}\sum_{i=1}^{q}\left(\left|S_{B}^{U}\left(x_{i}\right)\right|-\left|S_{B}^{U}\left(x_{i}\right)\cap S_{D}^{U}\left(x_{i}\right)\right|\right)+\frac{1}{{\left|U\right|}^{2}}\sum_{i=q+1}^{\left|U\right|}\left(\left|S_{B}^{U}\left(x_{i}\right)\right|-\left|S_{B}^{U}\left(x_{i}\right)\cap S_{D}^{U}\left(x_{i}\right)\right|\right)\\ \; & =\frac{1}{{\left|U\right|}^{2}}\sum_{i=1}^{q}\left(\left|S_{B}^{U}\left(x_{i}\right)\right|-\left|S_{B}^{U}\left(x_{i}\right)\right|\right)+\frac{1}{{\left|U\right|}^{2}}\sum_{i=q+1}^{\left|U\right|}\left(\left|S_{B}^{U}\left(x_{i}\right)\right|-\left|S_{B}^{U}\left(x_{i}\right)\cap S_{D}^{U}\left(x_{i}\right)\right|\right)\\ \; & =\frac{1}{{\left|U\right|}^{2}}\sum_{i=q+1}^{\left|U\right|}\left(\left|S_{B}^{U}\left(x_{i}\right)\right|-\left|S_{B}^{U}\left(x_{i}\right)\cap S_{D}^{U}\left(x_{i}\right)\right|\right) \end{array}$$

In addition, the following equation can be derived according to Lemma A3

$$\begin{array}{cc} \frac{1}{{\left|U\right|}^{2}}\sum_{i=q+1}^{\left|U\right|}\left(\left|S_{B}^{U}\left(x_{i}\right)\right|-\left|S_{B}^{U}\left(x_{i}\right)\cap S_{D}^{U}\left(x_{i}\right)\right|\right) & =\frac{{\left|U^{\prime }\right|}^{2}}{{\left|U\right|}^{2}}\frac{1}{{\left|U^{\prime }\right|}^{2}}\sum_{i=q+1}^{\left|U\right|}\left(\left|S_{B}^{U}\left(x_{i}\right)\right|-\left|S_{B}^{U}\left(x_{i}\right)\cap S_{D}^{U}\left(x_{i}\right)\right|\right)\\ \; & =\frac{{\left|U^{\prime }\right|}^{2}}{{\left|U\right|}^{2}}\frac{1}{{\left|U^{\prime }\right|}^{2}}\sum_{j=1}^{\left|U^{\prime }\right|}\left(\left|S_{B}^{U^{\prime }}\left(x_{j}\right)\right|-\left|S_{B}^{U^{\prime }}\left(x_{j}\right)\cap S_{D}^{U^{\prime }}\left(x_{j}\right)\right|\right)\\ \; & =\frac{{\left|U^{\prime }\right|}^{2}}{{\left|U\right|}^{2}}{\mathrm{EN}}^{U^{\prime }}\left(D\left|B\right.\right) \end{array}$$

Therefore, there exists

$$\begin{array}{c} \frac{{\mathrm{SIG}}_{2}^{\mathrm{outer}}\left(a,U,B,D\right)}{{\mathrm{SIG}}_{2}^{\mathrm{outer}}\left(a,U^{\prime },B,D\right)}=\frac{{\left|U^{\prime }\right|}^{2}}{{\left|U\right|}^{2}}. \end{array}$$

Hence, for ∀a,b∈C−B, if ${\mathrm{SIG}}_{2}^{\mathrm{outer}}\left(a,U,B,D\right)\geq {\mathrm{SIG}}_{2}^{\mathrm{outer}}\left(b,U,B,D\right)$ satisfies, then there exists ${\mathrm{SIG}}_{2}^{\mathrm{outer}}\left(a,U^{\prime },B,D\right)\geq {\mathrm{SIG}}_{2}^{\mathrm{outer}}\left(b,U^{\prime },B,D\right)$. □

**Theorem** **A3.**
*Let the pair $\left(U,C\cup D\right)$ be an IDT, such that we have B⊆C, $U^{\prime }=U-{\mathrm{POS}}_{B}^{U}\left(D\right)$ and β=0 satisfied. As for ∀a,b∈C−B, if ${\mathrm{SIG}}_{3}^{\mathrm{outer}}\left(a,U,B,D\right)\geq {\mathrm{SIG}}_{3}^{\mathrm{outer}}\left(b,U,B,D\right)$ satisfies, there exists ${\mathrm{SIG}}_{3}^{\mathrm{outer}}\left(a,U^{\prime },B,D\right)\geq {\mathrm{SIG}}_{3}^{\mathrm{outer}}\left(b,U^{\prime },B,D\right)$.*

**Proof.** Omitted because of its similarity to the proof of Theorem A1. □
